# Myoclonic seizure prior to diagnosis of chronic toxic encephalopathy: a case report

**DOI:** 10.1186/s13256-016-1188-9

**Published:** 2017-02-07

**Authors:** Wim L. C. Van Hooste

**Affiliations:** IDEWE Occupational Health Services – External Service for Prevention and Protection at Work, Interleuvenlaan 58, B-3001 Heverlee, Belgium

**Keywords:** Case report, Epilepsy, Occupational health, Organic solvents, Seizure, Thinner, Workplace, CTE, Chronic toxic encephalopathy

## Abstract

**Background:**

“Thinner” is a widely used industrial mixture of organic solvents. Exposure to organic solvents is usually not considered to be a possible cause of epilepsy, despite descriptions of toxic effects on the central nervous system. There are only a few reports about a possible epileptogenic effect of organic solvents exposure. We report a case of myoclonic seizure at the workplace that shows a remarkable coincidence between exposure to a thinner mixture and the occurrence of an epileptic seizure.

**Case presentation:**

We present the case of a 50-year-old Belgian woman exposed to organic solvents for more than 20 years in a paintbrush manufactory. In 2009, her biological monitoring of hippuric acid (primary urinary metabolite of toluene) exceeded the threshold limit value of the American Conference of Governmental Industrial Hygienists.

In 2012, after a period of high organic solvents exposure without the use of proper collective or personal airway protection, she had a seizure with myoclonic movements of her four limbs and loss of consciousness at her workplace. An electroencephalogram, computed tomography and magnetic resonance imaging of her head were within limits. Non-toxicological and toxicological causes were investigated. Her seizures did not reappear after workplace removal.

Two years after her epileptic insult she was diagnosed with chronic toxic encephalopathy type 2b.

In 2015, volatile organic compounds were measured at her workplace. Multiple (3/18) air samples exceeded the Belgian time-weighted average over 8 hours (77 mg/m^3^) for toluene.

**Conclusions:**

Knowledge about the impact of solvent exposure on the occurrence of epileptic insults is lacking. Long-term exposure to organic solvents is usually not considered to be a possible cause of epileptic seizure. But epileptic discharges have been described on electroencephalogram recordings for patients with chronic toxic encephalopathy. Myoclonic encephalopathy has been reported in toxic conditions.

This case emphasizes a possible unusual neurological presentation of occupational exposure to organic solvents. It may be explained by lowering of the threshold for seizures due to high solvents exposure, most probably by toluene. This case suggests a chronological connection between a high occupational exposure to solvents and an epileptic insult. We found no other plausible cause.

## Background

“Thinner” is a widely used industrial volatile liquid, a mixture of organic solvents (OS), used especially to thin paint. Toluene is the main component found in commercially available thinners. Exposure to OS is usually not considered to be a possible cause of epilepsy, despite descriptions of toxic effects on the central nervous system (CNS). We report a case of myoclonic seizure at the workplace that shows a remarkable coincidence between the exposure to an OS mixture and the occurrence of an epileptic seizure. Two years after the insult chronic toxic encephalopathy (CTE) was diagnosed.

## Case presentation

We present the case of a 50-year-old Belgian woman, occupationally exposed to OS for more than 20 years while employed at a paintbrush manufactory. At 18 years of age she was employed at a Belgian dry cleaning service for 6 years, without exposure to perchloroethylene (PER). From 26 years of age, her work tasks involved the use of OS mixtures and were performed without the use of proper collective or personal airway protection because her employer had neither invested in nor provided these preventive measures. In 2009, the biological monitoring of her hippuric acid in urine, the primary urinary metabolite of toluene, demonstrated a value (2540 mg/l) exceeding the threshold limit value (TLV) of 1600 mg/l of the American Conference of Governmental Industrial Hygienists [1]. In October 2012 her work task was removing paint from wrongly colored paintbrushes manufactured in China. Shortly after the start of this task, she began to complain of intense headaches, fatigue, asthenia, irritability, feeling of drunkenness, sleeping disturbances (sleepiness or, in contrast, difficulty in falling asleep), nausea, and paresthesia in her feet. She associated all of these classic symptoms of acute solvent intoxication with the use of the OS mixture, a thinner containing a high level of toluene, with which she worked permanently for over 2 months in a poorly ventilated work environment. Most symptoms disappeared during the weekend. She also had visual perception disturbances (scotomata). On a Thursday morning, 12 December 2012, she was very confused at her workplace and had aphasia. This was followed by involuntary myoclonic-type movements in all four limbs lasting for several minutes, she bit her tongue, and a loss of consciousness, possibly with prodromal aura phenomenon. After the seizures she was confused for a long time, with disorientation (postictal state). She was transported to a hospital. On admission, physical and neurologic examinations revealed no abnormalities. A blood count and blood biochemistry demonstrated normal liver and kidney tests, no elevated C-reactive protein, slightly elevated D-dimers, and elevated lactic acid. Unfortunately no toluene levels in blood were measured. An electrocardiogram (ECG) was sinusal. Her standard electroencephalogram (EEG) and brain computed tomography (CT) were within limits. Differentiating between cardiogenic or convulsive syncope and seizure is a diagnostic problem and challenge when involuntary movements (myoclonic jerks) are involved. In favor for the diagnosis of seizure were the episode duration of several minutes and that no arrhythmias were demonstrated. We found no arguments for triggered complex migraine and migralepsy before the seizure.

This event was her first epileptic insult. In her medical history we found a history of migraine, an extra-uterine pregnancy, female sterilization, and epicondylitis lateralis. Many etiological factors were investigated: non-toxicological and some toxicological (alcohol or any other addictive substances abuse) causes were excluded. She did not take or had not recently stopped any medication. There was no familial history of epilepsy. Further, there was no history of any disease prenatally, perinatally, or in childhood. She did not have and had not had any psychiatric illness, learning disability, attention deficit disorder, or a craniocerebral trauma.

She was observed at the hospital for 24 hours. No anti-epileptic drugs were started because of this first epileptic episode. Seizures did not reappear after work removal, although intoxication signs and symptoms remained.

A magnetic resonance imaging (MRI) of her brain performed approximately 2 months after the insult was within limits. A single-photon emission computed tomography (SPECT) of her brain did not demonstrate signs of Alzheimer disease or dementia.

Because some of her signs and symptoms (such as intense headaches, fatigue, asthenia, and irritability) continued in 2013, she was referred to a neurologist with expertise in CTE in 2014, who diagnosed that she had CTE type 2b. Later that same year, she developed a depression secondary to the diagnosis of CTE.

Tests for volatile organic compounds (VOC) at her workplace were consequently carried out on 19 November 2015. Three out of 18 air samples of different work task sites at the manufactory exceeded the Belgian time-weighted average (TWA; 8 hours) of 77 mg/m^3^ for toluene (90, 94, and 113 mg/m^3^). Measurements of 2-butanone, n-Butyl acetate, ethanol, isobutyl alcohol, and isopropyl alcohol did not exceed the Belgian TWA (8 hours).

## Discussion

Epilepsy is a condition affecting 2 to 5 % of the world’s population during their lifetime. Epilepsy is a disorder with many possible causes. Often a cause cannot be found. If there is an identifiable cause it usually involves some form of brain damage. Certain circumstances or substances can trigger a seizure: stress, lack of sleep, alcohol (binge drinking, hangover, withdrawal), illegal drugs, fever, or high temperature. In middle-aged persons epilepsy is most likely to be caused by strokes, tumors, or injuries. The convulsant threshold or seizure threshold is used to describe the balance between excitatory and inhibitory forces in the brain which affect the susceptibility of a person to seizures. A reduced seizure threshold is found in 10 % of the human population. This population is at higher risk of epileptic seizure in the case of exposure to harmful factors at the workplace or outside it [2]. Medications such as the antidepressant and nicotinic antagonist bupropion and the atypical opioid analgesics tramadol and tapentadol can lower the seizure threshold. So can other factors, including: sleep deprivation; fever; menstruation; uncontrolled diabetes; lengthy periods of fasting, malnutrition, starvation, high stress, fear, fatigue, and exhaustion; and exposure to flashing or flickering lights. Detection of the decreased convulsant threshold and gathering knowledge about potential epileptogenic factors at workplaces and in the community environment should become one of the goals for medical personnel dealing with occupational problems according to Stankiewicz and Stankiewicz [3].

There are a few reports about a possible epileptogenic effect of OS exposure [4–7]. Long-term exposure to OS is usually not considered a possible cause of epileptic seizure [8]. In 2002, Viaene reported that knowledge about the impact of solvent exposure on the occurrence of epileptic insults or the recurrence rate is lacking [9], although epileptic discharges have been described on EEG recordings for patients with CTE type 2b and type 3 [9], and for a worker chronically exposed to thinner [10]. Bernardini and Scoppetta [4] reported two cases among painters. The first patient had two generalized paroxysmal seizures during sleep. The second patient had acute solvent poisoning followed 7 weeks later with several epileptic episodes associated with typical EEG alterations. In both cases there was neither a history of neurological disease, nor any other neurological dysfunctions. The results of comprehensive neuroradiological studies were normal. Jacobsen *et al*. [5] reported the case of a painter chronically exposed to OS who had temporal epileptic seizures. Seizures disappeared after removal from work, and appeared again after exposure to cyclohexanone, although treated with anti-epileptic drugs. A case-referent study by Littorin *et al*. [6] on focal epilepsy has shown a just significant risk of the disease in individuals heavily exposed (painters) to OS.

Ginja *et al*. [7] reported a case of epilepsy in an employee exposed to PER. Disabling myoclonic encephalopathy has been reported in toxic conditions, for example after exposure to organic solvent trichloroethylene [9]. There are also reports of myoclonic encephalopathy in a series of toxic conditions due to: bismuth; methyl bromide; manganese; lead; mercury; colloidal silver; rodenticides containing alpha-chloralose; drugs such as dopa, lamotrigine, carisoprodol, cefuroxime and lithium; and mushroom ingestion [2].

Our literature review resulted in some animal studies [11–13] about seizures, convulsions, epilepsy, in association with exposure to solvents [4–8]. OS comprise chemically heterogeneous compounds, with a widespread application in a range of occupational settings and industries. Their volatility and lipophilicity make OS toxicologically important. OS rapidly contaminate the work environment and pose a major health risk in occupational settings. Solvents, or reactive metabolites, result in long-term health effects. Exposure mainly occurs through inhalation and skin contact. Owing to their lipophilic and hydrophilic properties, exposure affects the CNS. The neurotoxic effects are well known in occupational exposure occurring in poor industrial hygiene conditions [4] Toluene is the main agent found in commercially available thinners. Toluene is a hydrocarbon solvent that is insoluble in water. After inhalation it is absorbed by the lungs and bound to lipoproteins. The main toxic impact of toluene is on the CNS, probably explained by high cerebral perfusion and the affinity of toluene for lipid-rich tissues, from which it is slowly released [14]. The effect on the CNS may be depressant or excitatory, with euphoria in the induction phase followed by disorientation, tremulousness, mood lability, tinnitus, diplopia, hallucinations, dysarthria, ataxia, convulsions, and coma. A review by Yücel *et al*. [15] indicated that toluene preferentially affects white matter structures, periventricular regions, and subcortical regions.

More evidence suggests the possibility for some subcortical structures to initiate seizures independently. Findings from histopathologic, electrophysiologic, and brain imaging studies provide ample evidence demonstrating that like normal cerebral function, epileptic seizures involve widespread network interactions between cortical and subcortical structures. Different forms of generalized and focal epileptiform discharges and seizures engage various subcortical structures in varying ways [16].

## Conclusions

In conclusion, we have reported the case of a patient with a myoclonic seizure probably induced by OS poisoning. This case suggests a possible unusual neurological presentation of occupational exposure to OS. This case suggests a chronological connection between a high occupational exposure to solvents and an epileptic insult, but it is not possible to identify a definite causal relationship (anecdotal evidence because it is retrospective and not controlled) [17]. Animal data support the causal association of this biologically plausible event. We found no other plausible cause other than the acute and chronic high OS exposure.
